# Self-Diagnosis of Surgical Site Infections: Lessons from a tertiary care centre in Karachi, Pakistan

**DOI:** 10.12669/pjms.36.ICON-Suppl.1716

**Published:** 2020-01

**Authors:** Sana Z Sajun, Katherine Albutt, Umme Salama Moosajee, Gustaf Drevin, Swagoto Mukhopadhyay, Lubna Samad

**Affiliations:** 1 Sana Z Sajun, MSc. Interactive Research and Development, Karachi, Pakistan; 2 Katherine Albutt, MD MPH. Department of Surgery, Massachusetts General Hospital, Program in Global Surgery and Social Change, Harvard Medical School, Boston, USA; 3 Umme Salama Moosajee, BSc. Center for Essential Surgery and Acute Care, Global Health Directorate, Indus Health Network, Karachi, Pakistan; 4 Gustaf Drevin, Program in Global Surgery and Social Change, Harvard Medical School, Boston, USA; 5 Swagoto Mukhopadhyay, MD. Program in Global Surgery and Social Change, Harvard University, Department of Surgery, Boston, USA; 6 Lubna Samad, MRCS FCPS. Center for Essential Surgery and Acute Care, Global Health Directorate, Indus Health Network, Karachi, Pakistan

**Keywords:** Surgical Site Infection, Patient Self-screening, Low-and Middle-Income Countries, Pakistan

## Abstract

**Background and Objective:**

Surgical site infections (SSIs) usually manifest post-discharge, rendering accurate diagnosis and treatment challenging, thereby catalyzing the development of alternate strategies like self-monitored SSI surveillance. This study aimed to evaluate the diagnostic accuracy of patients and Infection Control Monitors (ICMs) to develop a replicable method of SSI-detection.

**Methods:**

A two-year prospective diagnostic accuracy study was conducted in Karachi, Pakistan between 2015 and 2017. Patients were educated about SSIs and provided with questionnaires to elicit symptoms of SSI during post-discharge self-screening. Results of patient’s self-screening and ICM evaluation at follow-ups were compared to surgeon evaluation.

**Results:**

A total of 348 patients completed the study, among whom 18 (5.5%) developed a SSI. Patient self-screening had a sensitivity of 39%, specificity of 95%, positive predictive value (PPV) of 28%, and negative predictive value (NPV) of 97%. ICM evaluation had a sensitivity of 82%, specificity of 99%, PPV of 82%, and NPV of 99%.

**Conclusion:**

Patients cannot self-diagnose a SSI reliably. However, diagnostic accuracy of ICMs is significantly higher and they may serve as a proxy for surgeons, thereby reducing the burden on specialized surgical workforce in LMICs. Regardless, supplementing post-discharge follow-up with patient self-screening could increase SSI-detection and reduce burden on health systems.

## INTRODUCTION

Surgical site infections (SSIs) are the most common hospital-acquired infection in low- and middle-income countries (LMICs), which are estimated to occur in one in every ten surgical patients^1^,^2^ contrary to an incidence rate of 2.6% and 2.9% in the USA and European countries respectively.^1^ In Pakistan, the best available evidence suggests that SSIs occur in between 4% and 12.5% of procedures.^3^,^4^ SSIs are a chief cause of morbidity and mortality that raise the cost of treatment for patients and tax already fragile health systems.^5^,^6^

Early detection and appropriate diagnosis is critical to reduce SSI-associated morbidity and mortality.^5^-^7^ Approximately, 84% of SSIs occur after a patient is discharged from the hospital, creating a host of surveillance and diagnostic challenges.^2^,^5^ These challenges are compounded in LMICs as patients are often lost to follow-up or are unable to travel long distances to seek care. Limitations of conventional methods of SSI-detection, coupled with the increasing importance being given to SSI incidence as a quality of care metrics have catalyzed the development of alternate surveillance strategies.

Hence, patient’s ability to self-detect SSIs post-discharge is increasingly being explored.^8^,^9^ Evidence from high-income countries (HICs) validates patients’ ability to diagnose wound complications with reasonable accuracy.^9^ Patient-based surveillance therefore denotes a cost-effective mode of post-discharge SSI-detection that alleviates the overworked healthcare providers. However, limited evidence is available from LMICs on self-monitored surveillance of SSIs.^1^

A patient-centered self-screening tool was developed to assess patients’ ability to accurately detect SSIs. The accuracy of patient self-screening and evaluation by Infection Control Monitors (ICMs) was analyzed to develop a simple, accurate, and reproducible method of SSI-detection.

## METHODS

A 24-month prospective diagnostic accuracy study was designed and implemented to evaluate the SSI self- and ICM-screening questionnaire between October 2015 and September 2017. The study was granted ethics approval by the Institutional Review Boards at The Indus Hospital (Interactive Research and Development IRB: IRD_IRB_2015_09_003) and the Boston Children’s Hospital- (IRB-P00020515).

The study was conducted at The Indus Hospital (TIH) - a 375-bed private tertiary care facility offering free-of-cost healthcare in the densely populated city of Karachi, Pakistan. TIH performs 6,000 operations annually for a nationwide catchment population, covering a variety of services. Surgical patients at TIH are scheduled for routine follow-up appointments at 14 and 30 post-operative days, during which a surgeon assesses overall recovery and identifies any post-operative complications.

All consecutive patients undergoing any of the 14 preselected surgical procedures (Table-I) at TIH were approached, consented and enrolled into the study. A priori sample size of 317 patients was calculated, using a web-based calculator,^10^ based upon an alpha of 0.01, power of 90%, assumed proportion of no event-event (no SSI by surgeon and SSI by patient or ICM) of 11%, proportion of event-no event (SSI by surgeon and no-SSI by patient or ICM) of 1.83% from the pilot study and an attrition rate of 30%.

**Table I:** Surgical procedures included in the study.

	Procedure	Total Patient Volume n (%)	SSI (n=18) n (%)	No SSI (n=330) n (%)	Chi-square p-value
Orthopedics	ORIF of Fracture	66 (18.9)	4 (22.2)	62 (18.8)	0.72
	Dynamic Hip Screw Arthoplasty	9 (2.6)	-	9 (2.7)	0.48
	Joint Replacement	3 (0.9)	-	3 (0.9)	0.68
General Surgery	Herniorraphy	70 (20.1)	4 (22.2)	66 (20.0)	0.82
Cardiothoracic	Coronary Artery Bypass Graft	33 (9.5)	3 (16.7)	30 (9.1)	0.29
Urology	Orchidopexy	3 (0.9)	-	3 (0.9)	0.68
	Cystolithotomy	1 (0.3)	-	1 (0.3)	0.82
Pediatric Surgery	ORIF of Fracture	14 (4.0)	2 (11.1)	12 (3.6)	0.12
	Herniorraphy	58 (16.7)	1 (5.6)	57 (17.3)	0.19
	Hydrocele Excision	1 (0.3)	-	1 (0.3)	0.82
	Cystolithotomy	1 (0.3)	-	1 (0.3)	0.82
	Orchidopexy	5 (1.4)	-	5 (1.5)	0.60
E.N.T	Thyroid Surgery	2 (0.6)	-	2 (0.6)	0.74
Ob/Gyn	Cesarean Section	82 (23.6)	4 (22.2)	78 (23.6)	0.89

**Fig.1:**
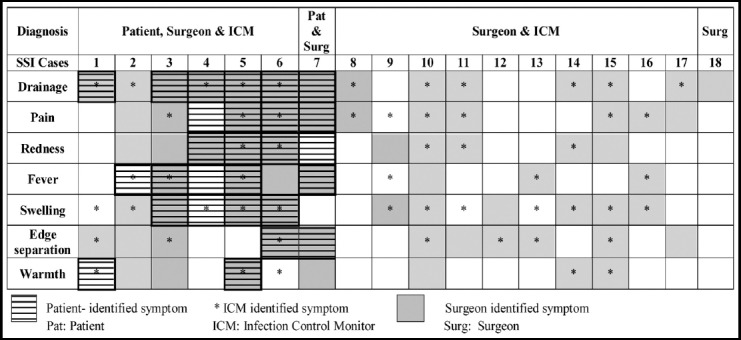
CDC Defined Symptoms Identification Matrix for true positive SSIs

An SSI was diagnosed and classified based upon the Center for Disease Control and Prevention (CDC) defined criteria i.e. “potential complications associated with a surgical procedure that typically occur at the surgical incision site within 30 post-operative days”.^11^ SSIs were diagnosed by a surgeon if at least one of the symptoms was present (Fig.1). Surgeon diagnosis of SSIs was considered as gold standard for comparison with other diagnoses. All surgeries were classified using CDC criteria as being clean, clean-contaminated, contaminated and dirty, depending on the extent of microbial contamination.^12^ Additionally, patients were categorized according to the WAMI Index, a proxy for socioeconomic status in LMICs.^13^

Moreover, an easy-to-use self-administered questionnaire was designed to elicit signs and symptoms of SSI through yes-or-no questions. This questionnaire was translated into Urdu, pre-tested on a small sample of patients and guardians and revised according to the feedback received. All enrolled patients received the questionnaire, a pictorial educational brochure on SSIs, and wound management counseling by one of the four designated ICMs, holding a degree in Bachelors of Nursing, post-surgery. Patients were advised to assess their surgical wounds at regular intervals post-discharge and were asked to fill the self-screening questionnaire at every screening. The signs and symptoms elicited from the questionnaire are detailed in Fig.1. Patients were advised to call the helpline immediately if they experienced any symptoms of SSI. Patients who called in with at least one positive symptom were scheduled for an appointment with the relevant surgical team within 24-48 hours. Rest of the patients attended follow-up appointments as planned.

Data were analyzed using SPSS version 21.0. Descriptive statistics were computed for each study variable. The sensitivity, specificity, positive predictive value (PPV), and negative predictive values (NPV) of patients’ and ICMs’ ability to detect SSIs compared with surgeons’ were calculated. Bivariate analysis was used to measure the association between any two variables of interest. Gwet’s AC1 was calculated to estimate the degree of inter-rater reliability in diagnosing a SSI.^14^ Additionally, logistic regression was performed to assess for a correlation between a patient’s ability to correctly self-screen for a SSI and their socio-economic status.

## RESULTS

### Demographics

A total of 454 patients consented and were enrolled into the study. Among these, 88 patients were lost to follow-up and 18 patients were excluded due to postponed surgical procedures or death unrelated to SSI, leaving a total of 348 who completed the study. Basic demographics of participants are presented (Table-II). A breakdown of study procedures is presented (Table-I). All surgeries were classified as “clean”.

**Table II:** Patient demographics.

		Total n (%)	SSI (n=18) n (%)	No SSI (n=338) n (%)	Chi-square p-value
Age	Mean ± SD	30.8 ± 19.4	37.8 ± 18.8	30.4 ± 19.4	0.12*
Gender	Female	139 (39.9)	8 (44.4)	131 (39.7)	0.81
	Male	209 (60.1)	10 (55.6)	199 (60.3)	
Marital Status	Single	116 (33.3)	3 (16.7)	113 (34.2)	0.20
	Married	232 (66.7)	15 (83.3)	217 (65.8)	
Area	Urban	328 (94.3)	16 (89.9)	312 (94.5)	0.28
	Rural	20 (5.7)	2 (11.1)	18 (5.5)	
Patient’s Primary Language	Urdu	243 (69.8)	10 (55.6)	233 (70.6)	0.20
	Sindhi	27 (7.8)	1 (5.6)	26 (7.9)	
	Punjabi	22 (6.3)	2 (11.1)	20 (6.1)	
	Pushto	24 (6.9)	1 (5.6)	23 (7.0)	
	Other	32 (9.2)	4 (22.2)	28 (8.5)	
Formal Education	Yes	224 (64.4)	12 (66.7)	212 (64.2)	1.00
	No	124 (35.6)	6 (33.3)	118 (35.8)	
Education Years†	Mean ± SD	4.8 ± 1.4	4.7 ± 1.1	4.8 ± 1.4	0.77*
WAMI Score‡	Mean ± SD	0.72 ± 0.1	0.75 ± 0.1	0.72 ± 0.1	0.22*

†Education Status of Caregiver/Parent was recorded for patients unable to read or write/pediatric patients

‡Water and sanitation, Assets, Maternal education, and monthly household Income (WAMI) Index; Ranges between 0-1

* t-test was used to calculate p-vales for Age, Education Years and WAMI Score.

### SSI Diagnosis

A total of 18 patients were diagnosed with SSIs, equating to a prevalence of 5.2%. Patients who developed an SSI did not differ from the rest in terms of demographic characteristics. There was no relationship between socioeconomic status, as measured by participants’ WAMI score, and presence or absence of SSI (p=0.22). There was no relationship between SSI diagnosis and surgical procedures.

### Self-Screening

Patients correctly reported an SSI in seven (38.9%) cases and correctly noted the absence of an SSI in 312 (94.5%) cases. In 11 (61.1%) cases, an infection was diagnosed by the surgeon during a routine post-operative visit but was not reported by the patient. In 18 (5.5%) cases, patients suspected a SSI but the surgeon did not confirm these findings (Table-III). The sensitivity of the screening tool was 38.9% (95% CI [18.3, 63.9]), specificity was 94.5% (95% CI [91.4, 96.6]), PPV was 28.0% (95% CI [12.9, 49.6]) and NPV was 96.6% (95% CI [93.8, 98.2]).

**Table III:** Patient and ICM Diagnoses vs. Surgeon Diagnosis.

		Patient self-screening n (%)		Total	ICM evaluation n (%)		Total

		SSI	No SSI	SSI	No SSI
Surgeon Diagnosis (n%)	SSI	7 (38.9)	11 (61.1)	18 (100.0)	14 (82.4)	3 (17.6)	17 (100.0)
	No SSI	18 (5.5)	312 (94.5)	330 (100.0)	3 (0.9)	327 (99.1)	330 (100.0)
Total		25 (7.2)	323 (92.8)	348 (100.0)	17 (4.9)	330 (95.1)	347 (100.0) †

†ICM results were calculated from a total of 347 observations; 1 case was omitted as the patient presented in the emergency room at The Indus Hospital out of study hours; as a result, a study ICM was not present at the time for evaluation.

### ICM Diagnosis

ICMs correctly reported a SSI in 14 (82.4%) cases and correctly noted the absence of a SSI in 327 (99.1%) cases. There were three false negative cases and three false positive cases (Table-III). The sensitivity of ICM evaluation was 82.4% (95% CI [55.8, 95.3]), specificity was 99.1% (95% CI [97.1, 99.8]), PPV was 82.4% (95% CI [55.8, 95.3]) and NPV was 99.1% (95% CI [97.1, 99.8]).

### SSI Symptoms

The most common signs/symptom used for SSI identification amongst patients was drainage (n=6, 85.7%) followed by pain at the incision site (n=4, 57.1%), redness (n=4, 57.1%), fever (n=4, 57.1%) and swelling (n=4, 57.1%). The most commonly documented symptoms amongst ICMs were swelling (n=11, 78.6%), drainage (n=10, 71.4%), pain (n=8, 57.1%), and wound separation (n=7, 50.0%). Surgeons detected drainage (n=13, 72.2%) most commonly, followed by pain (n=11, 61.1%), wound separation (n=11, 61.1%), redness (n=10, 55.6%), and swelling (n=10, 55.6%). There was no statistically significant difference between the symptoms used to identify an SSI and the type of identifier (p=0.99). Clustering of reported symptoms by patients, ICMs and surgeons, respectively is presented in a matrix (Fig.1).

## DISCUSSION

The SSI prevalence in this study was 5.2%, which is consistent with the existing estimates of SSI incidence in Pakistan i.e. between 4% and 12.5%.^3^,^4^ The relatively low rate of SSI could be attributed to all included surgeries being classified as “clean”.^2^-^4^ This rate is comparable to 4% SSI prevalence for “clean” cases in Zafar et al.’s multicenter study.^3^ It is important to note, regardless of low SSI prevalence, that SSI-associated morbidity and mortality are preventable making early detection and appropriate diagnosis essential to improving outcomes.^15^

In this study, patients were unable to accurately self-diagnose SSIs. With a self-screening sensitivity of only 38.9%, patients failed to diagnose SSIs in over half of the confirmed SSIs. Existing literature is highly variable in terms of the accuracy of patient self-screening.^8^,^16^ Studies that compared patient self-diagnosis with health professional diagnosis found substantial agreement between the two parties’ independent wound assessment with a few discordant assessments.

The variable results of self-screening have motivated some practitioners to explore techniques like telephone surveillance. Richter et al. found a sensitivity of only 66% in a meta-analysis of telephonic SSI surveillance.^16^ Others have suggested the supplementation of self-screening with photo documentation. For example, mPOWER uses Smartphone technology in the US to allow patients to share surgical wound photos and answer SSI triage questions during the post-discharge period.^17^ In LMICs, mobile and network requirements for effective Smartphone photo follow-up are a limiting factor in bringing such technology to scale, which may change in the coming years. A near perfect agreement was found between surgeon and ICM assessments at follow-up with high sensitivity and specificity, suggesting that ICMs can correctly diagnose SSIs. Other studies corroborate this finding suggesting that trained non-physicians can correctly identify SSIs and may be used as a proxy to surgeons for SSI-detection.^18^,^19^ In LMICs, task-shifting and task-sharing have been promoted as a response to the healthcare workforce crisis.^20^

Drainage was found to be the most frequent symptom identified by all cadres in true positive cases, which reinforces Whitby et al’s observation of discharge being the most common symptom used for SSI-detection by patients. However, drainage was often confused with serous discharge as opposed to infectious pus resulting in an overestimation of infection rates by the study patients.^8^ In our study, drainage was also identified in half of the false positive cases suggesting that the presence of drainage is not highly specific for SSIs. Despite extensive education regarding worrisome drainage characteristics, patients were unable to correctly identify pathologic wound drainage, resulting in over-identification of this symptom. Future patient-centered SSI interventions should emphasize the spectrum of discharge. Further research into constellation of signs and symptoms that best correlate with correct SSI diagnosis is necessary.

### Limitations of the study

First, the power to detect significant differences among groups was limited among this cohort owing to the low SSI prevalence rate. Second, the study failed to capture the true health-seeking pattern of patients by disregarding whether the patients self-screened at regular intervals using the questionnaire or if they self-screened but did not call in. Future interventions should include reminder systems to prompt patients to self-screen at regular intervals and encourage patients to come in for follow-up irrespective of SSI suspicion. Third, a 20% loss to follow-up rate made it impractical to consider ICM screening as the only form of SSI surveillance. Therefore, further research involving community health workers would help address this concern and provide an alternative to facility-based screening.

## CONCLUSION

This study employed a patient-centered tool for active post-discharge surveillance of SSIs in Pakistan. While patients were unable to accurately diagnose SSIs, ICMs may be used as a proxy to surgeons for SSI-detection, thereby reducing the burden on the specialized surgical workforce in LMICs. Supplementing regular post-discharge follow-up with ICM-screening in low-resource settings has the potential to increase the rate of SSI- detection with minimal additional burden to the health system.
